# Notes from Private Practice

**Published:** 1877-12

**Authors:** 


### NOTES FROM PRIVATE PRACTICE.

*Sulphate of Cinchonidia as an Anti-periodic.*

Any information which will tend to lessen a physician's expenditure for drugs, will, I take it, be thankfully received, especially in times such as these. I have recently noticed two or three articles with reference to substituting sulphate of cinchonidia for sulphate of quinia, the writers claiming that the cheaper drug fulfilled every indication met by quinine. I know that many physicians are not aware of this fact, and wish to add my testimony to that already given. I find that in malarial fevers of whatever type, the cinchonidia salt has proved just as certainly a specific, as the salt of quinia. Having had a large number of cases of this class, treated almost entirely with the drug in question, I have learned to place just as much confidence in it as I have had in quinine, and with equal confidence predict a favorable result. It has not failed in a single instance to prevent the next paroxysm in a tertian and the next but one, sometimes the next in a quotidian ague, and is equally efficacious in remittent fever, being well borne by the stomach, and not producing any of the unpleasant head symptoms which so certainly follow large doses of quinine. I administer it in five grain doses, either in pill or powder as the patient desires, every four hours, day and night, without any reference to paroxysm, intermission, remission or exacerbation, until the patient has passed safely through the "chill day," in a tertian ague, and through two days without chills in quotidian; then continue in smaller doses, say two grains after or before each meal. Considering the fact that quinine is sold at nearly four dollars per ounce and sulphate of cinchonidia at only eighty cents, every physician should be acquainted with the above facts. It will be seen that it only requires about a drachm (or ten cents worth) of the drug to completely control the disease.

WARREN Co., Illinois. H. L. HARRINGTON.

*Ingrowing Toe-nail.*

Among the various methods for treating ingrowing toe-nail, excepting when the toe is greatly inflamed, so as to necessitate removal of a portion of the nail, none has proved so satisfactory to me as the following:

The nail should be allowed to grow long—from three-sixteenths to one-fourth of an inch—on the affected side of the toe. The side of the nail should *not* be pared, but the hardened integuments should be removed from beneath its edge after soaking the toe in warm water. This being accomplished, a narrow strip of adhesive plaster should be slipped under the corner of the nail—adhesive side downward—and drawn firmly over the flesh which has been pressed up beside the edge of the nail, and fastened by both ends beneath the toe. This simple dressing can be renewed as often as necessary by the patient. As the length of the nail carries the corner beyond the toe, it cannot irritate the flesh and the pressure of the shoe upon the end of the nail tends to elevate its sides.

The plaster elevates the ingrowing portion of the nail and protects the tissue which had been irritated by it.

If, in addition to this treatment, the patient will discard tight shoes, he will be rewarded by a speedy and permanent cure.

Paring the side of the nail gives temporary relief, but insures the return of the trouble by leaving an irregular edge, and a sharp corner at the matrix, which must irritate as the nail grows.

Scraping the middle of the nail, and placing a little roll of cotton beneath its edge, if properly done, will sometimes give relief; but it seldom effects a cure.

Removal of a portion of the nail is painful and generally unnecessary. I have seen cases of long duration, in some of which suppuration had existed several months, speedily cured by the method here recommended, after all ordinary methods —save ablation—had been faithfully but unsuccessfully employed.

188 Clark St., Chicago. E. F. Ingals.

*Medicinal Condiments.*

The sodium-chloride which is found in all the tissues and fluids of the body, is so essential to osmosis and therefore to life, that it is taken into the body, in part at least, in the form of a pure chemical. What table is without its *salière?* The broil, the roast and the fry, the soup, the stew and the salad, are worse than useless without the addition of salt. A few years ago it was evident that my blood was not manufacturing normal hæmatin, and with a physician's dislike for medicine I became a subject of spanæmia. Confident there was something wrong with my assimilative powers, I sought for a remedy. The tincture of iron and the "elegant" elixir did not suit my taste, and therefore I put into my salt-cellar an equal quantity of sodium-chloride and Quevenne's iron. This compound, alimentary and tonic, was sprinkled upon my beefsteak and into my soup, and in brief time the improvement in my condition was pronounced. Not only sodium-chloride with iron, but other of the proximate principles—potash, magnesia, soda, lime, chlorides, phosphates, carbonates, etc.,—might be similarly employed. These, in varying quantities, combined with pharmaceutical exactness, and the proportion determined by the physician's knowledge of the particular constituents lacking in the disease or condition in question, might find their place, not in the vial, but in the *salt-cellar* to be used like pepper and salt with the food. Sometimes the fluids, sometimes the solids of the body suffer; now the bones, then the muscles, and the nervous tissues. These various indications may be at least approximately met by combinations of certain of the elementary substances of which the body is composed, administered in the form of *a condiment.*[1] Such a condiment may be prepared by thorough trituration to meet the conditions of a weakened digestion, for trituration adds much to the assimilability of a medicament. Such medicines (*food*) are most easily digested when the stomach, excited to secretion by the anticipation or presence of food, is ready to receive and dispose of them. It will be ob-

1 I do not claim that this is better than other modes, but that it meets certain individual caprices and idiosyncrasies.

served that I have conformed to common usage in the use of the word "medicine." Strictly speaking, iron and common salt are not medicines. They are related to physiology, not to pathology.

James I. Tucker, M. D.

